# Age-related changes in ultra-triathlon performances

**DOI:** 10.1186/2046-7648-1-5

**Published:** 2012-10-01

**Authors:** Beat Knechtle, Christoph Alexander Rüst, Patrizia Knechtle, Thomas Rosemann, Romuald Lepers

**Affiliations:** 1 Gesundheitszentrum St. Gallen, Vadianstrasse 26, St., Gallen, 9011, Switzerland; 2 Institute of General Practice and Health Services Research, University of Zurich, Rämistrasse 71, Zurich, 8006, Switzerland; 3 INSERM U1093, Faculty of Sport Sciences, University of Burgundy, Esplanade Erasme, Dijon, 21000, France

**Keywords:** Swimming, Cycling, Running, Ultra-endurance

## Abstract

**Background:**

The age-related decline in performance has been investigated in swimmers, runners and triathletes. No study has investigated the age-related performance decline in ultra-triathletes. The purpose of this study was to analyse the age-related declines in swimming, cycling, running and overall race time for both Triple Iron ultra-triathlon (11.4-km swimming, 540-km cycling and 126.6-km running) and Deca Iron ultra-triathlon (38-km swimming, 1,800-km cycling and 420-km running).

**Methods:**

The age and performances of 423 male Triple Iron ultra-triathletes and 119 male Deca Iron ultra-triathletes were analysed from 1992 to 2010 using regression analyses and ANOVA.

**Results:**

The mean age of the finishers was significantly higher for Deca Iron ultra-triathletes (41.3 ± 3.1 years) compared to a Triple Iron ultra-triathletes (38.5 ± 3.3 years) (*P* < 0.05). For both ultra-distances, the fastest overall race times were achieved between the ages of 25 and 44 years. Deca Iron ultra-triathletes achieved the same level of performance in swimming and cycling between 25 and 54 years of age.

**Conclusions:**

The magnitudes of age-related declines in performance in the three disciplines of ultra-triathlon differ slightly between Triple and Deca Iron ultra-triathlon. Although the ages of Triple Iron ultra-triathletes were on average younger compared to Deca Iron ultra-triathletes, the fastest race times were achieved between 25 and 44 years for both distances. Further studies should investigate the motivation and training of ultra-triathletes to gain better insights in ultra-triathlon performance.

## Background

In recent years, there has been an increased interest in investigating the effect of aging on endurance running performances [1-6]. Over the last decades, the participation of master athletes (>40 years old) has increased, especially in the longer run distances such as half marathons [2,3], marathons [1-3] and ultra-marathons [7-10]. However, with increasing age, the endurance performance decreases. In general, the peak endurance performance is maintained until the age of 30 to 35 years, followed by a moderate decline until the age of 50 to 60 years, and then a progressively steeper decline after the age of 70 to 75 years, independent of the length of the performance and the kind of the discipline [2-6,11-16].

Considering the age-related decline in male ultra-endurance athletes, Hoffman investigated ultra-marathoners competing over 161 kilometres [7-9]. Beyond the age of 30 to 39 years, the average finishing times increased linearly with increasing age. The 30- to 39-year-old males showed the fastest races times, with athletes in younger and older age groups being slower. The 40- to 49-year age group was approximately 4.0% slower than the 30 to 39 years one [8]. In another study of 161-km ultra-marathoners, performance of the athletes in the 40- to 49-year age group was no different from the performance of the athletes in the < 30 and the 30- to 39-year age groups [9]. Both the moderate decline in running performance and the large number of successful master athletes suggest that master athletes are able to maintain a high degree of physiological performance with increasing age [17]. For Ironman triathletes, both an increase in participation and an improvement in performance in master athletes have recently been reported [18,19].

The multi-sport discipline triathlon involves successively the three endurance disciplines: swimming, cycling and running. The traditional distances in triathlon vary from the short or Olympic distance, covering 1.5-km swimming, 40-km cycling and 10-km running [20-23], to the Ironman distance covering 3.8-km swimming, 180-km cycling and 42.2-km running [24,25]. Furthermore, ultra-endurance triathlons of longer distances than the Ironman distance do exist such as the Triple Iron ultra-triathlon of 11.4-km swimming, 540-km cycling and 126.6-km running [26,27], and the Deca Iron ultra-triathlon of 38-km swimming, 1,800-km cycling and 420-km running [28,29].

Regarding the performance in a triathlon, the association of different characteristics in physiology [30-33], anthropometry [34-37], training [34,35,38] and previous experience [28,36,37,39,40] with race time has been investigated. Apart from these characteristics, an age-related decline has been described for Olympic distance triathletes [20-23] as well as for Ironman triathletes [22,25]. It seems that the length of an endurance performance has an influence of the age-related performance decline. In short distance triathletes, a significant decline in performance starts at the age of 45 to 50 years [20,23]. In Ironman triathletes, however, the age-related decline in performance starts after the age of 55 years [22].

To date, no study has investigated the age-related decline in performance in an ultra-triathlon longer than the Ironman distance. Therefore, the aim of the present study was to analyse the age-related declines in swimming, cycling, running and total performances for both Triple Iron ultra-triathletes and Deca Iron ultra-triathletes. Since the age-related performance decline occurred later in the longer Ironman triathlons than in the shorter Olympic distance triathlon, we hypothesised that the age-related decline in performance would occur even later for Deca Iron ultra-triathletes than for Triple Iron ultra-triathletes.

## Results and Discussion

### Participation trends

From 1992 to 2010, there were 423 Triple Iron ultra-triathlon finishers and 119 Deca Iron ultra-triathlon finishers. During this period, the average number of finishers per year was 23 ± 9 (range, 7 to 38) at the Triple Iron ultra-triathlon and 11 ± 5 (range, 2 to 18) at the Deca Iron ultra-triathlon, respectively. From 1992 to 2010, the number of total finishers per age group, for both races, is shown in Table 1. The 5-year age bracket with the largest participation was 35 to 39 years in the Triple Iron ultra-triathlon and 40 to 44 years in the Deca Iron ultra-triathlon.

**Table 1:** Total number of finishers per age group in both the Triple and Deca Iron ultra-triathlons (1992 to 2010)

**Iron distance**	**Age groups (years)**								
	**25 to 29**	**30 to 34**	**35 to 39**	**40 to 44**	**45 to 49**	**50 to 54**	**55 to 59**	**60 to 64**	**65 to 69**
Triple	12	93	127	102	41	29	12	6	1
Deca	3	19	29	38	11	9	7	2	1

### Age of the ultra-triathletes

**Figure 1:**
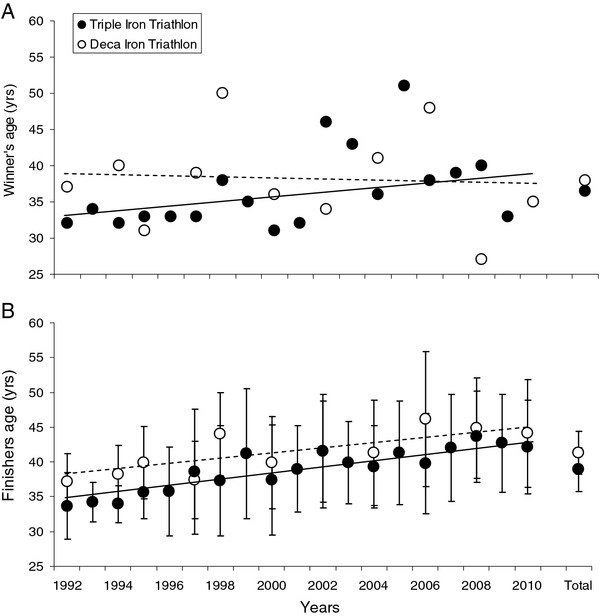
**Ages of the winners both in the Triple and Deca Iron triathlons.** Covering from 1992 to 2010 (**A**). Mean (±SD) age of the finishers in the Triple and Deca Iron triathlons from 1992 to 2010 (**B**). The years analysed are pooled, and the mean values are shown on the right side of the panels (Total). The dashed lines represent the linear regressions for the Deca Iron triathletes, and the continuous lines represent the linear regressions for the Triple Iron triathletes between 1992 and 2010. For the winners’ age, the gradients were not different from zero for both the Deca (*r*^2^ = 0.01, *P* = 0.07, *y* = −0.141x + 319.9) and Triple Iron triathletes (*r*^2^ = 0.18, *P* = 0.07, *y* = 0.409x − 781.4). In contrast, the age of the finisher gradients increased by 0.4 years per annum (*r*^2^ = 0.66, *P* = 0.002, *y* = 0.426x − 811.1) for the Deca Iron triathletes and by 0.5 years per annum (*r*^2^ = 0.83, *P* = 0.0001, *y* = 0.506x − 974.7) for the Triple Iron triathletes.

The ages of the winners from 1992 to 2010, for both the Triple Iron ultra-triathletes and Deca Iron ultra-triathletes, are shown in Figure 1A. The ages of the winners did not significantly change over this period, in either of these races and, therefore, no differences in the mean age of the winners was found: 35.8 ± 4.5 years (range, 31 to 46 years) in the Triple Iron ultra-triathlon and 38.0 ± 6.8 years (range, 27 to 50 years) in the Deca Iron ultra-triathlon. In contrast, the mean age of the finishers was significantly (*P* < 0.05) higher in the Deca Iron ultra-triathlon compared with the Triple Iron ultra-triathlon; 41.3 ± 3.1 years in the Deca Iron ultra-triathlon and 38.5 ± 3.3 years in the Triple Iron ultra-triathlon (Figure 1B). In addition, the mean age of the finishers significantly increased over this period for both ultra-triathlon distances.

### Age-related changes in performance

Total, swimming, cycling and running performances of the top ten triathletes for the different age groups at the Triple Iron triathlon during the 1992 to 2010 period are shown in Table 2. There was a significant age effect for swimming (*F* = 62.4, *P* < 0.01), cycling (*F* = 65.7, *P* < 0.01), running (*F* = 209.9, *P* < 0.01) and the total (*F* = 158, *P* < 0.01) times. No significant difference in time was observed between the age groups 25 to 34 and 35 to 44 years for swimming, cycling, running and total time. The mean best time performances were 176 ± 7 min for swimming (25 to 34 years), 1,030 ± 24 min for cycling (35 to 44 years), 759 ± 38 min for running (35 to 44 years) and 2,069 ± 92 min for the total race time (35 to 44 years).

**Table 2:** Total, swimming, cycling and running performances of the top ten Triple Iron ultra-triathletes

**Age groups (years)**		**Total time (min)**	**11.4-km swim time (min)**	**540-km cycle time (min)**	**126-km run time (min)**
25 to 34	Mean	2,091	176	1,037	803
	SD	33	7	27	35
35 to 44	Mean	2,069	180	1,030	759
	SD	92	3	24	38
45 to 54	Mean	2,288^a^	198^a^	1,124^a^	909^a^
	SD	55	6	50	34
55 to 64	Mean	2,828^a,b^	235^a,b^	1,287^a,b^	1,230^a,b^
	SD	137	19	70	69

These are the performances of the different age groups at the Triple Iron ultra-triathlon (data were pooled from 1992 to 2010). ^a^Significantly different (*P* < 0.01) from age groups 25 to 34 years and 35 to 44 years. ^b^Significantly different (*P* < 0.01) from age groups 25 to 34 years, 35 to 44 years and 45 to 54 years.

Total, swimming, cycling and running performances of the top ten triathletes for the different age groups at the Deca Iron triathlon during the 1992 to 2010 period are shown in Table 3. There was a significant age effect for swimming (*F* = 32.2, *P* < 0.01), cycling (*F* = 28.1, *P* < 0.01), running (*F* = 12.7, *P* < 0.01) and the total (*F* = 22.2, *P* < 0.01) times. No significant difference in running and total times was observed between the age groups 25 to 34 and 35 to 44 years. No significant differences in times were observed for the age groups between 25 and 54 years for swimming and cycling. The mean best time performances were 732 ± 41 min for swimming (35 to 44 years), 5,758 ± 247 min for cycling (35 to 44 years), 5,055 ± 807 min for running (25 to 34 years) and 12,292 ± 616 min for total performances (35 to 44 years), respectively.

**Table 3:** Total, swimming, cycling, and running performances of the top 10 Deca Iron triathletes

**Age groups**		**Total time (min)**	**38-km swim time (min)**	**1,800-km cycle time (min)**	**420-km run time (min)**
25 to 34 years	Mean	12,954	816	6,403	5,055
	SD	788	91	568	807
35 to 44 years	Mean	12,292	732	5,758	5,134
	SD	616	41	247	388
45 to 54 years	Mean	14,177^a^	855	6,514	6,426^b^
	SD	1,053	81	469	647
55 to 64 years	Mean	16,405^c^	1,213^c^	8,245^c^	6,996^b^
	SD	1,948	194	1,004	1,300

These are the performances of the different age groups at the Deca Iron triathlon (Data were pooled from 1992 to 2010). ^a^Significantly different (*P* < 0.01) from age groups 35 to 44 years. ^b^Significantly different (*P* < 0.01) from age groups 25 to 34 years and 35 to 44 years. ^c^Significantly different (*P* < 0.01) from age groups 25 to 34 years, 35 to 44 years and 45 to 4 years.

The aim of this study was to analyse the age-related declines in swimming, cycling, running and total performances in male Triple and Deca Iron ultra-triathletes. No difference in total performance was found between the ages of 25 and 44 years for both Triple and Deca Iron ultra-triathletes. However, in contrast to Triple Iron ultra-triathletes, Deca Iron ultra-triathletes can achieve the same level of performance in swimming and cycling between 25 and 54 years of age.

### The effects of age on the different disciplines of triathlon

A main interesting finding was that the age-related decline occurred later in swimming and cycling compared to running at the Deca Iron ultra-triathlon distance. Previous studies suggested that the age-related decline in triathlon performance was specific to the discipline, with cycling showing fewer declines in performance with age than running in both short distance and Ironman triathletes [20,22]. The present results showed that the swimming and cycling performances were maintained until the age of 54 for the Deca Iron ultra-triathletes but only until the age of 44 for the Triple Iron ultra-triathletes. A potential explanation for this finding could be pre-race experience. It has been shown that previous experience was a strong predictor for a successful finish in an ultra-triathlon [28,39]. Recently, Lepers et al. [41] analysed the performance of 73 triathletes (68 men and 5 women) who finished a Double Iron ultra-triathlon, a Triple Iron ultra-triathlon and a Deca Iron ultra-triathlon. The contribution of swimming to overall ultra-triathlon performance was lower than for cycling and running. Running performance was more important to overall performance for Double Iron ultra-triathlon and Triple Iron ultra-triathlon compared to Deca Iron ultra-triathlon. The Double Iron ultra-triathlon and Triple Iron ultra-triathlon performances were significantly correlated to Deca Iron ultra-triathlon performances for swimming and cycling, but not for running.

The main differences between cycling and running are the change from a nonweight to a weight-bearing activity and the differences in the coordination of the leg muscles with a shift from a predominantly concentric type of muscle action in cycling to a stretch-shortening activity with eccentric contractions in running [42]. Cycling may show differences in the age-related performance decline compared to running due to the contraction types involved. Cycling as a nonweight-bearing activity uses predominantly concentric muscle activation compared with the stretch-shortening activity and eccentric activations during running. As older adults show different rates of decline during fatiguing contractions that involve eccentric as opposed to concentric activations, this may explain the differences in the rate of decline in cycling versus running [43].

### The best age to perform in ultra-triathlons

Since the age-related performance started to decline later in an Ironman distance triathlon than in an Olympic distance triathlon, we hypothesised that the age-related decline in performance would occur later for Deca Iron ultra-triathletes than for Triple Iron ultra-triathletes and the fastest performance in a Deca Iron ultra-triathlon would be achieved at a higher age than in a Triple Iron ultra-triathlon. In contrast to our hypothesis, the age of the best overall performance did not differ between Triple Iron ultra-triathlon and Deca Iron ultra-triathlon and was comprised between 25 and 44 years for both ultra-triathlon distances. However, the group with the largest participation at the Triple Iron ultra-triathlon (35 to 39 years) was younger compared to the Deca Iron ultra-triathletes (40 to 44 years). We analysed in the present study the age-related decline in Triple Iron ultra-triathletes competing for approximately 2,000 min compared to Deca Iron ultra-triathletes racing for approximately 12,000 min. A significant age effect was found for total race time with no difference between the distances. A previous study evidenced that the age-related decline in total performance was less pronounced for the Olympic distance triathlon (approximately 2 to 3 h) than for the Ironman distance triathlon (approximately 9 to 15 h) [22].

The present data showed that, in ultra-triathlons, the athletes were able to maintain their best performances for ages comprised between 25 and 44 years, independent of the distance. In half-marathoners [2], marathoners [2] and short distance triathletes [20-23], generally athletes in the age group 20 to 30 years showed the fastest race times compared to athletes in the other age groups. For longer distances such as the Ironman triathlon, athletes in the age group 30 to 39 years were faster compared to athletes in the age group 20 to 29 years [25]. Apart from training, also the aspect of pre-race experience seems to be important for a successful finish in an ultra-triathlon. It has been demonstrated that the personal best time in a Triple Iron triathlon, not anthropometry or training volume, was associated with total race time in a Triple Iron triathlon [39]. Furthermore, the number of finished Triple Iron triathlons and the personal best time in a Triple Iron triathlon, but not anthropometry, were also related to Deca Iron ultra-triathlon race time [28].

**Figure 2:**
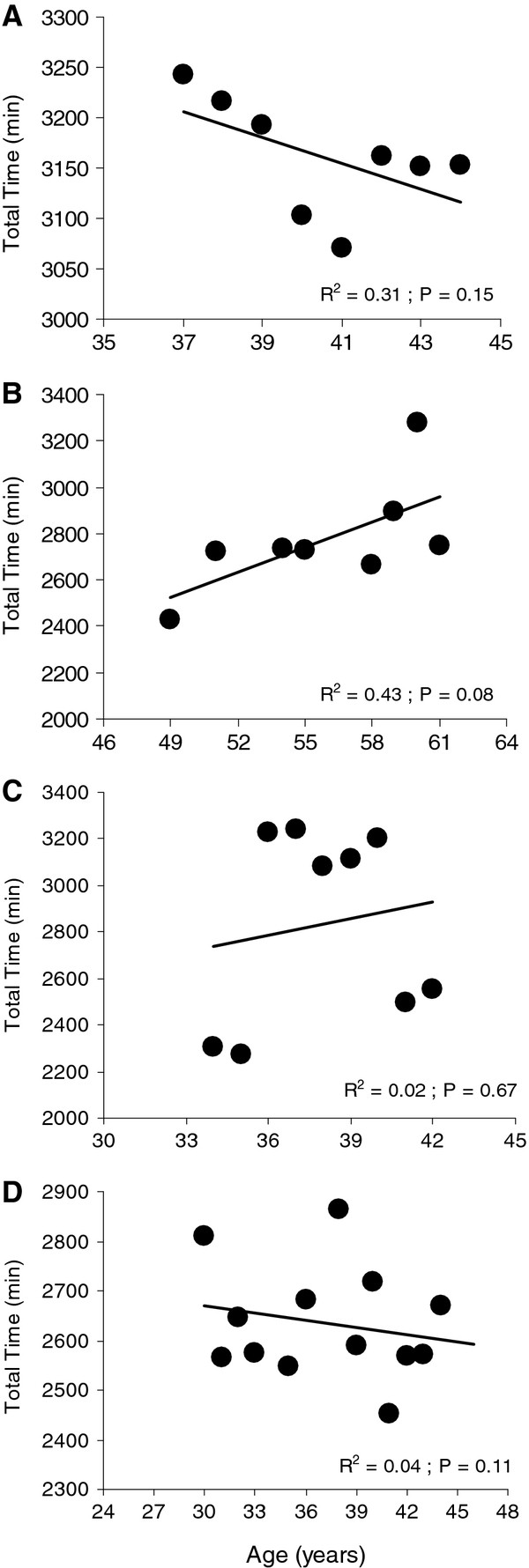
**Change in total performance at the Triple Iron ultra-triathlon for four ultra-triathletes.** The event was held in Lensahn, Germany. These athletes (**A**, **B**, **C** and **D**) finished at least eight times the event between 1992 and 2010. For all four triathletes, their performances did not significantly change across the age.

It would be interesting in future studies to perform a longitudinal observation and to compare the age-related decline from cross-sectional and longitudinal data. A short analysis of the present data showed that four ultra-triathletes finished at least eight times the Triple Iron ultra-triathlon between 1992 and 2010. For these four ultra-triathletes, their total performances did not significantly change across the ages (Figure 2). These findings suggest that some exceptional ultra-triathletes are able to perform at the same level of performance during an 8- (subject A) to 16-year (subject D) period.

### Physiological considerations

Tanaka and Seals [4] demonstrated that the changes in endurance performance with aging were attributed to reductions in VO_2_max and the decline in lactate threshold. Findings for competitive long-distance runners indicated that the decline in running times parallel the age-related reductions in VO_2_max and in lactate threshold [15]. For runners, mean VO_2_max declined from 71.4 ml · min^−1^ · kg^−1^ in youth to 41.8 ml · min^−1^ · kg^−1^ at a mean age of 56.6 years [44]. The decrease in an ultra-endurance performance may be attributed to an age-related loss in skeletal muscle mass. A large component of lost independence with increasing age is weakness due to a loss of lean muscle mass. Muscle power is lost at a greater rate than is endurance capacity with 3.5% versus 1.8% per year, respectively [6]. The age-related loss of muscle mass is primarily due to a decrease in the size of type II (fast-twitch) muscle fibres and a change in the proportions of fibre types [45,46]. There is an increase in the proportion of type II fibres, which may adapt to high endurance demands [47].

The age-related decline was more pronounced in running compared to cycling for both the Triple Iron ultra-triathletes and the Deca Iron ultra-triathletes. Running involves stretch-shortening cycles with eccentric muscle actions, while cycling is a nonweight-bearing activity with predominant concentric muscle actions. With increasing age, the fast-twitch muscle fibres atrophy more than slow-twitch muscle fibres [48]. Since the fast-twitch fibres seem to be more susceptible to damage than the slow-twitch fibres during stretch-shortening cycles, the greater reduction in running performance compared with cycling could, therefore, be related to muscle typology changes with increasing age [49]. However, the changes in muscle fibre type distribution such as the percentage of type I muscle fibres with advancing age seem to be less pronounced in well-trained master athletes compared to untrained older adults [5].

A further aspect in these ultra-endurance races is the loss of substantial body mass such as skeletal muscle mass and fat mass. In ultra-endurance races, a loss in body mass has been described, contributing to a decrease in solid masses. An ultra-endurance performance leads to a decrease in both skeletal muscle [50-54] and fat masses [55-57]. In Ironman [50], Triple Iron ultra-triathletes [26,55], and Deca Iron ultra-triathletes [28], a significant decrease in skeletal muscle mass has been described. It is, therefore, likely that the more pronounced age-related decline in running performance in the Deca Iron ultra-triathletes was rather due to the considerably longer nature of the race with the corresponding loss in skeletal muscle mass than the effect of age. The decrease in skeletal muscle mass is most probably due to the increased mechanical stress due to the eccentric nature of running. It has been demonstrated that longer distance running in an ultra-marathon induced more impact-stress on the skeletal muscle than a marathon [58].

### Limitations of the present study

The first limitation of this cross-sectional study is the problem arising from the data collection. Firstly, it is not sure at 100% that there are no data errors (age, race time) when taking them from the race websites and the official rankings. Secondly, we cannot assure that the age-related performance decline is not at least partly due to non-participation of older athletes or simply to a self-chosen lower intensity of older athletes. In addition, race participants may represent selected sub-groups of the total population of ultra-triathletes, e.g. some potential participants may have been prevented from participating due to many reasons.

It was not possible to collect other performance-related factors such as characteristics of training [36,37,39], anthropometry [27,29,34,35,39] and previous experience [28,37,38]. Thus, these factors had to be ignored in this study. In addition, we have not included environmental conditions. It has been shown that an ultra-performance [74,75] progressively slows down when the ambient temperature increases [59,60].

## Conclusions

This cross sectional study found that the magnitudes of age-related declines in ultra-triathlon performance for Triple Iron and Deca Iron triathlon are quite similar. For both Triple Iron and the Deca Iron ultra-triathletes, the fastest race times were achieved between 25 and 44 years of age, although participants at the Triple Iron triathlon were on average younger compared to the Deca Iron triathlon. Further studies should investigate the motivation and training of ultra-triathletes to gain better insights in ultra-triathlon performance.

### Availability of supporting data

The data sets supporting the results of this article are available in http://www.triathlonlensahn.de and http://www.multisport.com.mx.

## Methods

This study was approved by the Institutional Review Board of St. Gallen, Switzerland, with a waiver for the requirement of an informed consent given by the subjects since the study involved the analysis of publicly available data. For two annual ultra-triathlons, one Triple Iron triathlon and one Deca Iron triathlon, the age of the athlete in the year of the race together with the swimming, cycling, running and total race times in the year of each race were analysed from 1992 to 2010. During this period, both the Triple Iron triathlons in Lensahn, Germany and the Deca Iron triathlons in Monterrey, Mexico were held regularly. The data set from this study was obtained from the race websites http://www.triathlonlensahn.de for the Triple Iron triathlon Germany in Lensahn, http://www.multisport.com.mx for the Deca Iron triathlon in Monterrey, Mexico and from the race directors involved.

### Races

The Triple Iron ultra-triathlon in Germany took place in Lensahn, Schleswig-Holstein, Germany, and was comprised of 11.6-km swimming, 540-km cycling and 126.6-km running. The swimming was held in a 50-m heated outdoor pool at a temperature of approximately 25°C, and wetsuits were allowed. After passing through the transition area, the participants cycled 67 laps of 8 km each on a hilly course in the surroundings of the town. At the next transition, the athletes changed and ran 96 laps of a 1.31-km per lap flat run course in the town of Lensahn. The cycling course was nearly free of road traffic, and the run course was completely free of traffic and illuminated during the night. All the athletes had their own support crew to provide nutrition and changes of clothes or equipment. The athletes had to arrive at the finish line within 58 h of the race start. The Deca Iron ultra-triathlon took place in Monterrey, Mexico. The athletes completed a total distance of 38-km swimming, 1,800-km cycling and 422-km running. Swimming commenced in the 50-m outdoor pool in Monterrey in the ‘Sociedad Cuauhtemoc & Famosa Park’, 3 km away from the cycle and run track in ‘Parque Niños Héroes’. The pool was not heated and the water temperature was approximately 27°C. The laps of 100 m were counted by personal lap counters for each athlete. After completing the swimming, the athletes changed in the transition area, and due to the high traffic volume, were transferred by car to ‘Parque Niños Héroes’. A period of 30 min was allowed for the transfer from pool to park, which was deducted from the final race time. The park was closed to traffic, completely illuminated and with a 1.915-km cycle/run track that is approximately 95% flat but included an inclination of approximately 5%. After the cycling, the athletes changed and went directly to the run course which was on the same track but in the opposite direction. Drafting in the cycle section was strictly prohibited and controlled by the race director. The laps on both the cycle and run courses were counted electronically using a microchip system. The athletes can be helped by their own support crew for nutrition and changes of equipment and clothes. During the whole race, accommodation is offered in the Sports Village inside the park, about 250 m away from the race site. The athletes and their support crews have a room with bed, toilet and shower. For nutrition, the organiser offered a variety of food in a restaurant, at the race site, that was open 24 h a day.

### Data analysis

#### Age of the participants

In each race year, the age of the winner and the mean age of the male finishers were analysed at both the Triple Iron ultra-triathlons and the Deca Iron ultra-triathlons from 1992 to 2010.

#### Age-related changes in performance

In order to analyse the age-related changes in swimming, cycling, running and the total race performances, we pooled the data from 1992 to 2010 for both distances. First, in order to evaluate the participation across the ages, we distinguished each age group category as follows: 25 to 29 years, 30 to 34 years, 35 to 39 years, 40 to 44 years, 45 to 49 years, 50 to 54 years, 55 to 59 years, 60 to 64 years and 65 to 69 years, respectively. Because of the small number of subjects per age group, we secondly considered only 4 age-groups for the analysis of performances: 25 to 34 years, 35 to 44 years, 45 to 54 years and 55 to 64 years, respectively. Performance times were converted to minutes. For the Triple and Deca Iron ultra-triathlons, the fastest ten swimming, cycling and running times, together with the total event times for each age group were determined for the 19-year period.

#### Statistical analysis

Data are reported as means ± standard deviations (±SD) in the text and the figures. Linear regressions were used for estimating the changes in the age of winners and the mean age of finishers *per year*. Pearson’s correlation coefficients were used to assess the association between the age and the years. One-way ANOVA was used to compare the ages of the Triple and the Deca Iron ultra-triathlon winners and the mean ages of the finishers. One-way ANOVA was used to compare the swimming, cycling, running and total race times between the different age groups for both distances. Tukey’s post hoc analyses were used to test differences within the ANOVA when appropriate. Statistical significance was accepted at *p* < 0.05 (Statsoft, Version 6.1, Statistica, Tulsa, OK, USA).

## Competing interests

The authors have no conflict of interest.

## Authors’ contributions

KB collected the data and drafted the manuscript. RCA added statistical analyses and helped draft the manuscript. KP collected the data. RT participated in the study design and helped draft the manuscript. LR performed the statistical analyses and helped draft the manuscript. All authors read and approved the final manuscript.
